# Adverse outcome pathway development from protein alkylation to liver fibrosis

**DOI:** 10.1007/s00204-016-1814-8

**Published:** 2016-08-19

**Authors:** Tomislav Horvat, Brigitte Landesmann, Alfonso Lostia, Mathieu Vinken, Sharon Munn, Maurice Whelan

**Affiliations:** 1 Chemicals Safety and Alternative Methods Unit (F.3), Directorate F – Health, Consumers and Reference Materials, Directorate General Joint Research Centre, European Commission, Ispra, Italy; 2 Department of In Vitro Toxicology and Dermato-Cosmetology, Center for Pharmaceutical Research, Vrije Universiteit Brussel (VUB), Brussels, Belgium

**Keywords:** Adverse outcome pathway (AOP), Liver fibrosis, Alternatives to animal testing, Risk assessment, Systems toxicology

## Abstract

In modern toxicology, substantial efforts are undertaken to develop alternative solutions for in vivo toxicity testing. The adverse outcome pathway (AOP) concept could facilitate knowledge-based safety assessment of chemicals that does not rely exclusively on in vivo toxicity testing. The construction of an AOP is based on understanding toxicological processes at different levels of biological organisation. Here, we present the developed AOP for liver fibrosis and demonstrate a linkage between hepatic injury caused by chemical protein alkylation and the formation of liver fibrosis, supported by coherent and consistent scientific data. This long-term process, in which inflammation, tissue destruction, and repair occur simultaneously, results from the complex interplay between various hepatic cell types, receptors, and signalling pathways. Due to the complexity of the process, an adequate liver fibrosis cell model for in vitro evaluation of a chemical’s fibrogenic potential is not yet available. Liver fibrosis poses an important human health issue that is also relevant for regulatory purposes. An AOP described with enough mechanistic detail might support chemical risk assessment by indicating early markers for downstream events and thus facilitating the development of an in vitro testing strategy. With this work, we demonstrate how the AOP framework can support the assembly and coherent display of distributed mechanistic information from the literature to support the use of alternative approaches for prediction of toxicity. This AOP was developed according to the guidance document on developing and assessing AOPs and its supplement, the users’ handbook, issued by the Organisation for Economic Co-operation and Development.

## Introduction

Liver fibrosis typically results from repeated-dose toxic injury and is an important human health issue associated with chemical exposure, which disrupts the normal liver architecture, alters organ function and may further develop to cirrhosis and liver cancer with considerable mortality attributable to these end stages (Bataller and Brenner 2005; Carey and Carey 2010; Lee 2003; Lim and Kim 2008; Mehta et al. 2014; Ramachandran and Kakar 2009). Regardless of the causing stimuli (i.e. toxic, metabolic, inflammatory, parasitic, or vascular), chronic hepatic injury may lead to liver fibrosis by the same mechanisms (Friedman 2003). This pathophysiological response not only affects the liver, but is common to many organs and tissues, where chronic injury triggers a series of key events (KEs) that finally leads to fibrosis. Same factors, as described in this work, contribute synergistically to fibrogenesis (Chen and Raghunath 2009; Johnson and DiPietro 2013; Kisseleva and Brenner 2008; Liedtke et al. 2013; Wynn 2008).

Animal models, especially rodents, are commonly used to study cellular and molecular mediators of fibrosis. In addition, human health chemical risk assessment is based on whole animal toxicity testing with single chemicals of concern (OECD 2013). Ethical considerations and international animal welfare rules, as well as the uncertain predictability of animal testing for human adverse health effects, represent limiting factors for in vivo testing (O’Brien et al. 2006; Seok et al. 2013). Moreover, due to costs and time involved, it is not feasible to use these methods for testing all of the chemicals that could affect human health (Krewski et al. 2010; OECD 2013). “Toxicity testing in the twenty-first century” aims to understand the underlying mechanisms of toxicity, rather than rely on direct observations of toxic effects. Sufficiently perturbed cellular response pathways can turn into “toxicity pathways” that result in adverse health effects (Berg et al. 2011; Kavlock et al. 2009; Krewski et al. 2010). In this context, the adverse outcome pathway (AOP) conceptual framework was developed as a tool for supporting chemical risk assessment based on mechanistic reasoning.

**Fig. 1:**
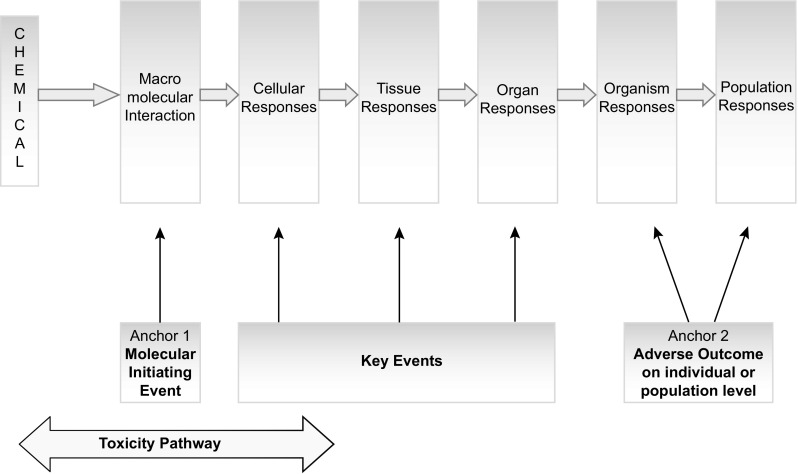
A schematic representation of the adverse outcome pathway (AOP). An AOP starts with a molecular initiating event in which a chemical interacts with a biological target (*anchor 1*) leading to a sequential series of intermediate key events at different levels of biological organisation to produce an adverse outcome with relevance to risk assessment (*anchor 2*)

Conceptually, an AOP can be viewed as a sequence of events starting with an initial interaction of a stressor with a biomolecule in a target cell—termed the molecular initiating event (MIE)—and progressing through a dependent series of intermediate KEs at different levels of biological organisation, finally culminating in an adverse outcome (AO). AOPs are typically represented sequentially, moving from one KE to another, as compensatory mechanisms and feedback loops are overcome. These KEs are a limited number of measurable and toxicologically relevant molecular occurrences that are essential for progression towards the AO. It is important to keep in mind that an AOP does not provide a comprehensive molecular description of every aspect of the biology involved (the mechanism of action), but focuses on the critical steps in the pathway (Fig. 1) (Ankley et al. 2010; OECD 2013). The structured mechanistic knowledge enables development of integrated testing strategies, which rely on using in vitro methods, preferably based on human cells or human cell constituents, that in combination with in silico approaches facilitate in vivo predictions of toxicity and chemical risk assessment (Berg et al. 2011; Krewski et al. 2010; Vinken 2013).

Since liver fibrosis results from a complex interplay between various hepatic cell types, receptors and signalling pathways (Cong et al. 2012) elaborate multi-cell models for the investigation of toxicological processes in vitro are required. Liver slices, hepatic cell lines, and primary hepatocytes are currently the leading models for in vitro liver toxicity testing; embryonic or induced pluripotent stem cells are being introduced as renewable source of cells. The selection of liver models is increasing and novel cell culturing strategies such as three-dimensional cell culturing systems and co-cultures with two or more liver cell types are being devised. However, these models still lack the ability to functionally express the in vivo-like phenotype over long periods (Godoy et al. 2013; LeCluyse et al. 2012; Soldatow et al. 2013; Van de Bovenkamp et al. 2007; Westra et al. 2014).

Here, we describe the AOP developed for liver fibrosis, from hepatic injury caused by protein alkylation to the formation of liver fibrosis. It is among the first AOPs that were developed according to the guidance document on developing and assessing AOPs and its supplement, the users’ handbook, issued by the Organisation for Economic Co-operation and Development (OECD). The AOP development activity was started in the context of the Safety Evaluation Ultimately Replacing Animal Testing (SEURAT-1, http://www.seurat-1.eu) research project, which aimed at developing alternative models for safety assessment based on mechanistic knowledge. To this aim, AOPs for chronic liver injury were developed in order to facilitate design of studies for predicting selected types of repeated-dose toxicity (Landesmann et al. 2012; Vinken 2015; Vinken et al. 2013). Along with the ongoing evolution of the AOP concept, this pathway description has been revised, extended, and modified within the AOP Wiki (https://aopkb.org/aopwiki/index.php/Main_Page), a central AOP development platform and repository within the OECD AOP Development Work Plan, managed by the Extended Advisory Group on Molecular Screening and Toxicogenomics (http://www.oecd.org/chemicalsafety/testing/adverse-outcome-pathways-molecular-screening-and-toxicogenomics.htm). The AOP Wiki is one component of the AOP Knowledge Base (https://aopkb.org) that has been created to enable the scientific community, in one central location, to share, develop, and discuss their AOP-related knowledge.

## Basic principles of AOP development

Building an AOP requires describing a sequence of events from a MIE to an AO, while establishing causal links between individual KEs. By definition, AOPs are not chemical-specific and the pathway description should be independent from any specific chemical initiator (Villeneuve et al. 2014). Nevertheless, in the context of a particular AO, experimental data derived from exposure to prototypic chemicals are useful for understanding the patterns of biological response. Therefore, an AOP development process starts by identifying the compounds that have been clinically proven to be inducing the particular adversity of interest. Development of the AOP described here was based on two SEURAT-1 reference chemicals for liver fibrosis, namely carbon tetrachloride (CCl_4_) and allyl alcohol (Jennings et al. 2014), with their common MIE being protein alkylation (covalent protein binding reaction). By selecting first the AO followed by progressively tracing down molecular response to lower levels of biological organisation, in order to connect that outcome with a specific molecular initiating event, we applied a top-down strategy for AOP development. Understanding normal physiological processes is the basis for describing the perturbations that occur following chemical exposure. KEs, defined as changes in biological state, which have to be both measurable and essential for the specific AO, were analysed according to different levels of biological organisation. However, the choice of the relevant level of detail in AOP description is crucial. Indeed, too many details might distract from understanding the main pathway while being too concise holds the risk of overlooking relevant processes. Other than the KEs, key event relationships (KER) constitute a major feature added to the initial AOP reporting format. A KER is a scientifically based relationship that defines the connection between two KEs, by identifying one as an upstream and the other one as a downstream event. As such, it facilitates inference or extrapolation of the state of the downstream KE from the known, measured, or predicted state of the upstream KE (Villeneuve et al. 2014).

**Fig. 2:**
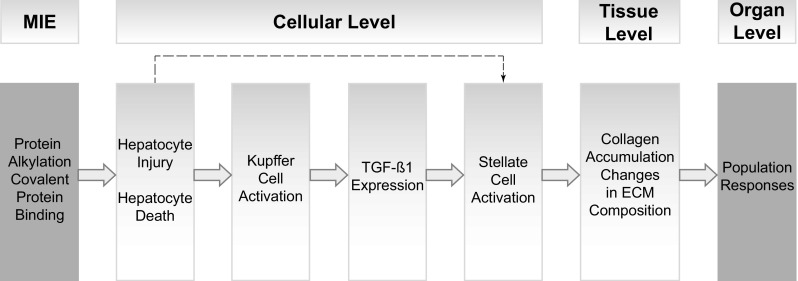
Graphic representation of the adverse outcome pathway from protein alkylation to liver fibrosis. The molecular initiating event (MIE) is protein alkylation, leading to structural and functional cell injury and cell death, the first key event (KE). Injured and apoptotic hepatocytes activate Kupffer cells, the next KE along the pathway. Activated KCs are the main source of TGF-β1, the most potent pro-fibrogenic cytokine. TGF-β1 expression causes the next KE, stellate cell activation, which leads to progressive collagen accumulation that together with changes in extracellular matrix (ECM) composition signifies the KE on tissue level. Collagen bands progress further to bridging fibrosis, finally affecting the whole organ. Full arrows represent direct KERs that link two adjacent KEs. The *dotted line* represents an indirect KER that bridges some of the KEs in the pathway

**Fig. 3:**
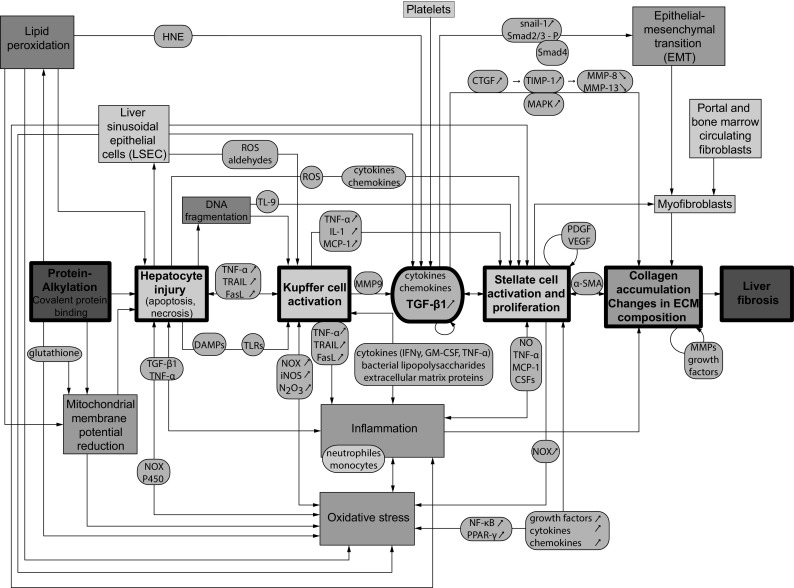
Network of molecular events triggered during development of liver fibrosis. Molecular mechanisms, feed-back, and feed-forward loops as well as inter-relationships between individual key events are presented. The *central line* of events, marked by *thick black frames*, represents the developed AOP, as shown in Fig. 2. *Violet boxes* correspond to MIE and AO, *blue boxes* to molecular processes, and *green boxes* to various cell types involved in fibrogenesis. *Orange ovals* represent molecular mediators. *α-SMA* alpha-smooth muscle actin, *CTGF* connective tissue growth factor, *DAMPs* damage-associated molecular patterns, *FasL* Fas Ligand, *GM-CSF* granulocyte macrophage colony-stimulating factor, *HNE-4* hydroxynonenal, *IFNγ* interferon gamma, *iNOS* nitric oxide synthase, *MAPK* mitogen-activated protein kinases, *MCP-1* monocyte chemoattractant protein-1, *MMPs* metalloproteinases, *N*_*2*_ *O*_*3*_ peroxinitrite, *NF-κB* nuclear factor kappaB, *NO* nitric oxide, *NOX* NADH oxidase, *P450* cytochrome P450, *PDGF* platelet-derived growth factor, *PPARγ* peroxisome proliferator-activated receptor-gamma, *ROS* reactive oxygen species, *CSFs* colony-stimulating factors, *TGF-β1* Transforming growth factor beta1, *TIMP-1* tissue inhibitor of metalloproteinases-1, *TNFα* tumor necrosis factor alpha, *TLRs* toll-like receptors, *TRAIL* TNF-related apoptosis-inducing ligand, and *VEGF* vascular endothelial growth factor (colour figure online)

AOPs are simplified pragmatic constructs, defined as linear, non-branching, and directed sequences of KEs, connecting a single MIE to an AO. The challenge lies in finding an acceptable linear graphical AOP representation (Fig. 2) in spite of the various existing feed-back and feed-forward loops and inter-relationships between KEs (Fig. 3). Linear AOPs are at the basis of the weight of evidence (WoE) evaluation (Villeneuve et al. 2014), done according to Bradford Hill considerations (Meek et al. 2014) and following the AOP Handbook (OECD 2014). The overall assessment of an AOP is best supported by thorough descriptions of KEs and their interrelationships (KERs), also demonstrating essentiality, biological plausibility, empirical support, and consistency of supporting data. Essentiality of KEs is demonstrated by studies in which blocking a KE prevents the occurrence of a further downstream KEs and ultimately the AO. Table 1 shows the overall assessment of the level of confidence in the overall AOP based on essentiality of KEs and biological plausibility, as well as empirical support for KERs. We present the AOP for liver fibrosis following the temporal occurrence of the individual AOP components.

**Table 1:** Overall assessment of the weight of evidence supporting the AOP based on essentiality of key events (KEs) and biological plausibility, as well as empirical support for key event relationships (KERs)

KE	KE description	Support for essentiality of the KE
		Defining question: are downstream KEs and/or the AO prevented if an upstream KE is blocked?
MIE protein alkylation	Covalent protein alkylation by reactive electrophiles was identified as a key triggering event in chemical-induced toxicity	
KE 1 hepatocyte injury/death	Covalent binding to liver proteins and oxidative stress can directly affect the cell or influence signalling pathways, finally leading to necrotic or apoptotic cell death	Essentiality of KE 1 is high
		Pharmacological inhibition of liver cell apoptosis attenuates liver injury and fibrosis suggesting a critical role for hepatocyte apoptosis in the initiation of HSC activation and hepatic fibrogenesis (Canbay et al. 2004a, b)
KE 2 Kupffer cell (KC) activation and macrophage recruitment	Activated KCs are a major source of inflammatory mediators and a main source of transforming growth factor beta 1 (TGF-β1)	Essentiality of KE 2 is high
		Pretreatment with gadolinium chloride, which inhibits KC function, reduced both hepatocyte and LSEC injury, as well as decreased the numbers of macrophages appearing in hepatic lesions, and inhibited TGF-β1 mRNA expression in macrophages (Andres et al. 2003; Ide et al. 2005). Experimental inhibition of KC function or depletion of KCs appeared to protect against chemical-induced liver injury (Lotersztajn et al. 2005; Schumann et al. 2000)
KE 3 TGF-β 1 expression	TGF-β1 is the most potent profibrogenic cytokine and plays a central role in fibrogenesis	Essentiality of KE 3 is high
		Animal experiments using different strategies to block TGF-β1 have demonstrated significant antifibrotic effects for liver fibrosis. Experimental fibrosis can be inhibited by anti-TGF-β treatments with neutralising antibodies or soluble TbRs (TGF-β receptors) (Cheng et al. 2009; Liu et al. 2006; Lotersztajn et al. 2005; Qi et al. 1999; Tang et al. 2012; Westra et al. 2014)
KE 4 hepatic stellate cell (HSC) activation	HSC activation signifies the transdifferentiation from a quiescent vitamin A–storing cell to a proliferative and contractile myofibroblast, the central effector in hepatic fibrosis	Essentiality of KE 4 is high
		Experimental inhibition of HSC activation prevents fibrosis (Nakamura et al. 2014; Son et al. 2009). Antifibrotic therapeutic strategies include inhibition of HSC proliferation or stimulation of HSC apoptosis (Anan et al. 2006; Li et al. 2008; Lotersztajn et al. 2005)
KE 5 collagen accumulation	Excess ECM (extracellular matrix) deposition and changes in ECM composition	Essentiality of KE 5 is high
		Continuing imbalance between the deposition and degradation of extracellular matrix is a prerequisite for liver fibrosis (Bataller and Brenner 2005; Lee and Friedman 2011)
Adverse Outcome liver fibrosis	Excessive deposition of ECM proteins occurs as a result of repeated cycles of hepatocytes injury and repair and results in liver fibrosis	

Event	Description	
Chronic inflammation	Hepatic fibrosis is commonly preceded by inflammation and persistent inflammation has been associated with progressive liver fibrosis. The fibrinogenic cascade is maintained by inflammatory mediators and inflammatory and fibrogenic cells stimulate each other in amplifying fibrosis. Damaged hepatocytes, KCs, HSCs, all release inflammatory cytokines that further activate fibroblastic cells (Fujiwara and Kobayashi 2005)	Studies have indicated that sustained suppression of inflammatory activity or dampening the immune response can halt and even reverse the fibrotic process (Czaja 2014). Studies examining the role of individual inflammatory cell populations in experimental models give evidence that the immune system can regulate both the progression and the regression of liver fibrosis (Pellicoro et al. 2014)
Oxidative stress	Oxidative stress-related molecules modulate tissue and cellular events responsible for the progression of liver fibrosis (Kirkham 2007; Parola and Robino 2001; Poli 2000; Singh and Czaja 2007)	Antioxidants display antifibrogenic properties in cell cultures and in experimental animal models (Lotersztajn et al. 2005). Specifically plateletderived growth factor (PDGF) -induced increase in collagen deposition and liver fibrosis is markedly reduced by treatment with the anti-oxidant MnTBAP chloride, a cell permeable superoxide dismutase (SOD) mimetic and peroxynitrite scavenger (El Rigal et al. 2013)

KER	Support for biological plausibility of the KER	Empirical support for the KER
	Defining question: Is there a mechanistic (i.e. structural or functional) relationship between KE_up_ and KE_down_ consistent with established biological knowledge?	Defining question: Does the empirical evidence support that a change in KE_up_ leads to an appropriate change in KE_down_? Does KE_up_ occur at lower doses and earlier time points than KE_down_ and is the incidence of KE_up_ > than that for KE_down_?
MIE to KE 1	Biological Plausibility is high Hepatocytes are damaged by alkylating agents via both covalent binding to liver proteins and lipid peroxidation accompanied by oxidative stress and collapse of mitochondrial membrane potential which triggers cell death (Codreanu et al. 2014; Kaplowitz 2002; Tanel and Averill-Bates 2007)	Empirical Support is moderate There is some experimental evidence that covalent protein alkylation does lead to cell injury, but neither targeted biomolecules nor threshold values have been identified yet (Bauman et al. 2009; Thompson and Burcham 2008)
KE 1 to KE 2	Biological plausibility is high KCs are activated upon engulfment of hepatocyte apoptotic bodies and through reactive oxygen species (ROS), cytokines and chemokines, which are released by damaged hepatocytes (Guo and Friedman 2010; Jaeschke 2011; Orrenius et al. 2011; Ramaiah and Jaeschke 2007)	Empirical support is moderate There is some experimental evidence that engulfment of hepatocyte apoptotic bodies by KCs stimulated the generation of cytokines (Canbay et al. 2004a, b; Luckey and Petersen 2001; Poli 2000; Takehara et al. 2004; Tukov et al. 2006)
KE 1 to KE 4	Biological plausibility is high HSCs are activated by damaged hepatocytes by similar mechanisms as KCs (Canbay et al. 2004a, b; Friedman 2008; Kisseleva and Brenner 2008; Lee and Friedman 2011; Li et al. 2008; Malhi et al. 2010; Roth et al. 1998; Zhan et al. 2006)	Empirical support is moderate It has been experimentally demonstrated that inhibition of liver cell apoptosis leads to significantly reduced markers of HSC activation and collagen I expression (Canbay et al. 2002, 2004a, b). Furthermore, it was observed that hepatocyte-HSC co-culturing increased the secretion of pro-inflammatory cytokines (Coulouarn et al. 2012)
KE 2 to KE 3	Biological plausibility is high Following activation KCs become a main source of TGF-β1, inflammatory mediators, and ROS (Bataller and Brenner 2005; Brenner 2009; Guo and Friedman 2007; Kirkham 2007; Kolios et al. 2006; Lee and Friedman 2011)	Empirical support is moderate Cytokine release is one of the features that define KC activation and there is empirical evidence for this KER (Chu et al. 2013; Matsuoka and Tsukamoto 1990)
KE 3 to KE 4	Biological plausibility is high TGF-β1 activates HSCs, i.e. stimulates cell proliferation, matrix synthesis, and release of retinoids by HSCs and is the most potent fibrogenic factor for HSCs (Bataller and Brenner 2005; Brenner 2009; Friedman 2002, 2008; Gressner et al. 2002; Kisseleva and Brenner 2007; Li et al. 2008; Liu et al. 2006; Parsons et al. 2007)	Empirical support is moderate HSCs can be activated by TGF-β1 in culture. However, HSCs activated in vitro do not fully reproduce the changes in gene expression observed in vivo (De Minicis et al. 2007). Czaja et al. (1989) did prove that treatment of cultured hepatic cells with TGF-β1 increased type I pro-collagen mRNA levels 13-fold due to posttranscriptional gene regulation. Tan et al. (2013) discovered that short TGF-β1 pulses can exert long-lasting effects on fibroblasts. Accumulated CD11b1 macrophages are critical for activating HSCs (via expression of TGF-β1) (Chu et al. 2013)
KE 4 to KE 5	Biological plausibility is high It is general accepted knowledge that activated HSCs are the primary collagen producing cell, the key cellular mediators of liver fibrosis (Benyon and Arthur 2001; Lee and Friedman 2011; Li et al. 2008; Milani et al. 1994)	Empirical support is moderate Analytical methods in vitro focus on measurement of pro-collagen secreted into culture medium or measurement of α-smooth muscle actin (α-SMA) expression, a marker of fibroblast activation (Brenner 2009; Rockey et al. 1992). In primary culture, HSCs from normal liver begin to express α-SMA coincident with culture-induced activation (Chen and Raghunath 2009; Yin et al. 2013)
KE 5 to AO	Biological Plausibility is high By definition liver fibrosis is the excessive accumulation of ECM proteins, which leads to disruption of normal hepatic architecture (Lee and Friedman 2011)	Empirical Support is high There is a smooth transition from ECM accumulation to liver fibrosis without a definite threshold and plenty in vivo evidence exists that ECM accumulation is a pre-stage of liver fibrosis (Bataller and Brenner 2005; Brancatelli et al. 2009; Pellicoro et al. 2014; Poynard et al. 1997; Rockey and Friedman 2006)

## MIE: protein alkylation

The initial chemical–biological interaction of this AOP for liver fibrosis is protein alkylation, which is the addition of an alkyl group (i.e. derived from an alkane following removal of one hydrogen atom) to a protein amino acid. Alkylating agents are highly reactive chemicals that introduce alkyl radicals into biologically active molecules and as a consequence can impede or alter their biological function (Liebler 2008; Russmann et al. 2009). Covalent protein alkylation by reactive electrophiles was identified as a major triggering event in chemical toxicity more than 40 years ago (Codreanu et al. 2014; Kehrer and Biswal 2000).

### KER between protein alkylation and hepatocyte injury/death

Even though protein alkylation is a generic process having an impact on multiple physiological processes in the cell, there is experimental evidence that covalent protein alkylation does lead to cell injury (Bauman et al. 2009; Thompson and Burcham 2008). Alkylated proteins can disturb the cellular redox balance in exposed cells by interacting with glutathione, which leads to a disruption of a plethora of biochemical pathways and intracellular stress that, depending on the extent of mitochondrial involvement, can lead to apoptotic or necrotic cell death (Codreanu et al. 2014; Kaplowitz 2002; Tanel and Averill-Bates 2007). Indeed, allyl alcohol/acrolein is considered a mitochondrial toxin that leads to cell death (Moghe et al. 2015). Whether apoptosis or necrosis ensues after acrolein exposure appears to be related to dose and cell type. In regards to activation of caspases as part of the mitochondrial death pathway, it was shown that apoptosis in human cells is caspase-dependent, as demonstrated in human neuroblastoma cells (Dong et al. 2013) and in A549 lung cells (Roy et al. 2009). It was suggested that the activation of certain caspases may arise from a partial inhibition of their active site cysteine residue through direct alkylation by acrolein (Kern and Kehrer 2002). Furthermore, using biotin hydrazide labelling, it was shown that NF-κB RelA and p50, as well as JNK2, were revealed as direct targets for alkylation by acrolein, affecting the GSH depletion. Mass spectrometry analysis of acrolein-modified recombinant JNK2 indicated adduction to Cys(41) and Cys(177), putative important sites involved in mitogen-activated protein kinase (MAPK) kinase (MEK) binding and JNK2 phosphorylation (Hristova et al. 2012). In complimentary work, exposure of cultured hepatocytes to acrolein led to a sustained activation of ERK1/2, JNK, and p38, which was associated with ER and mitochondrial stress and apoptosis. The cytotoxic effects of acrolein were decreased by JNK inhibitor, suggesting that kinase activation may be linked to cell death and liver injury (Mohammad et al. 2012). Finally, lipid peroxidation accompanied by oxidative stress and collapse of mitochondrial membrane potential can as well trigger apoptotic cell death (Fig. 3) (Kehrer and Biswal 2000; Liebler 2008; Manibusan et al. 2007).

## KE: hepatocyte injury/death

Chemicals and their metabolites can exert their effects either directly on cellular macromolecules (i.e. proteins, lipids and DNA) in mitochondria, cytoskeletal components, endoplasmic reticulum and nucleus, or indirectly through activation or inhibition of signalling cascades and transcription factors as well as changes in transcriptional activity. The outcome may be either triggering of necrotic or apoptotic processes or sensitisation for the action of cytokines (Kaplowitz 2002; Malhi et al. 2010; Orrenius et al. 2011). Today, hepatocyte injury is considered as essential for triggering fibrogenesis with hepatocellular apoptosis being increasingly viewed as a nexus between liver injury and fibrosis (Johnson and DiPietro 2013; Canbay et al. 2002, 2004a, b; Lotersztajn et al. 2005). Pharmacological inhibition of liver cell apoptosis attenuates liver injury and fibrosis suggesting a critical role for hepatocyte apoptosis in the initiation of hepatic stellate cell activation and hepatic fibrogenesis (Canbay et al. 2004a, b).

### KER between hepatocyte injury/death and Kupffer cell (KC) activation

Damaged hepatocytes can trigger KC activation via diverse molecular pathways. They release reactive oxygen species (ROS), cytokines, including transforming growth factor beta 1 (TGF-β1) and tumor necrosis factor alpha (TNF-α), and chemokines which all contribute to oxidative stress, inflammatory signalling and finally activation of KCs (Orrenius et al. 2011). ROS generation in hepatocytes results from oxidative metabolism by NADH oxidase (NOX) and cytochrome P450 2E1 activation as well as through lipid peroxidation. Damaged liver cells also trigger a sterile inflammatory response with activation of innate immune cells through release of damage-associated molecular patterns (DAMPs), which activate KCs through toll-like receptors (TLRs) and recruit activated neutrophils and monocytes into the liver. Central to this inflammatory response is the promotion of ROS formation by these phagocytes (Jaeschke 2011; Guo and Friedman 2010; Ramaiah and Jaeschke 2007). In addition, apoptotic hepatocytes can undergo genomic DNA fragmentation and formation of apoptotic bodies. These apoptotic bodies are consecutively engulfed by KCs thereby causing their activation (Canbay et al. 2003a, b, 2004a, b; Liu et al. 2010). The increased phagocytic activity strongly up-regulates NOX expression in KCs, a superoxide producing enzyme with pro-fibrogenic activity, as well as nitric oxide synthase (iNOS) mRNA levels, followed by a consequent harmful reaction between ROS and nitricoxide (NO) that leads to generation of cytotoxic peroxinitrite (N_2_O_3_) (Paik et al. 2014). ROS and/or diffusible aldehydes are also derived from liver sinusoidal endothelial cells (LSECs) which are additional initial triggers of KC activation (Poli 2000) (Fig. 3). Experiments on cells of the macrophage lineage showed significant aldehyde-induced stimulation of protein kinase C activity, an enzyme involved in several signal transduction pathways. Furthermore, aldehydic products of lipid peroxidation, such as 4-hydroxynonenal (HNE), were demonstrated to up-regulate TGF-β1 expression and synthesis in isolated rat KCs (Luckey and Petersen 2001).

Complete understanding of the hepatocyte–KC interaction and of its consequences for both normal and toxicant-driven liver responses still remains a challenge. KC activation followed by cytokine release is associated in some cases with evident liver damage, whereas in other cases this event is unrelated to liver damage or may be even protective. Apparently, the impact is dependent on the extent of KC activation, whereby excessive or prolonged release of KC mediators can switch from an initially protective mechanism to a damaging inflammatory response. Evidence suggests that low levels of cytokine release from KCs constitute a survival signal that protects hepatocytes from cell death and in some cases, stimulates proliferation (Kisseleva and Brenner 2008; Kolios et al. 2006; Malhi et al. 2010). Therefore, this KER is biologically plausible, but empirical evidence is currently rather limited. Unfortunately, activation of KC does not result in morphological changes, so staining techniques cannot be employed. A more reliable marker is cytokine release, even though it has to be evaluated in view of KCs’ propensity to activate spontaneously in in vitro conditions. Indeed, addition of KCs to hepatocytes in vitro does mimic drug-induced inflammatory responses in vivo (Tukov et al. 2006). Nevertheless, it was experimentally proven that engulfment of hepatocyte apoptotic bodies stimulated KCs to generate cytokines (Canbay et al. 2004a, b; Luckey and Petersen 2001). Takehara et al. (2004) showed that persistent apoptosis of parenchymal cells led to increased TGF-β production and consecutive development of liver fibrosis in vivo, as well as increased TGF-β expression by macrophages in vitro.

### KER between hepatocyte injury/death and hepatic stellate cell (HSC) activation

In addition to KC activation, damaged hepatocytes can also lead to activation of HSCs though the release of ROS, cytokines, and chemokines. Engulfment of apoptotic bodies from hepatocytes can result in activation and induction of NOX expression in HSCs (Paik et al. 2014; Zhan et al. 2006). DNA from apoptotic hepatocytes induces TL 9—dependent changes of HSCs that are consistent with late stages of HSC differentiation (activation), with up-regulation of collagen production and inhibition of platelet-derived growth factor (PDGF)-mediated chemotaxis to retain HSCs at sites of cellular apoptosis. The release of latent TGF-β complex into the microenvironment by damaged hepatocytes is likely to be one of the first signals for adjacent HSCs leading to their activation (Canbay et al. 2004a, b; Friedman 2008; Kisseleva and Brenner 2008; Lee and Friedman 2011; Li et al. 2008; Malhi et al. 2010; Roth et al. 1998) (Fig. 3).

This KER describes the linkage between two non-adjacent KEs and is, therefore, called indirect. It is biologically plausible and there is experimental evidence to demonstrate a mechanistic link between hepatocyte apoptosis and fibrogenesis. Markers of HSC activation were significantly reduced after pharmacological inhibition of liver cell apoptosis using a pan-caspase inhibitor (Canbay et al. 2002, 2004a, b). Furthermore, it was observed that hepatocyte-HSC co-culturing increased the secretion of pro-inflammatory cytokines (Coulouarn et al. 2012). Fluorescently labelled hepatocyte apoptotic bodies were added to cultures of primary and immortalised human HSCs that readily engulfed apoptotic bodies in a time-dependent manner, followed by an increase in alpha-smooth muscle actin (α-SMA) (primary cells), TGF-β1, and collagen alpha1(I) mRNA (primary and immortalised cells). It was shown that pro-fibrogenic response was dependent upon apoptotic body engulfment, since nocodazole, a microtubule-inhibiting agent, blocked both the engulfment and the increase of TGF-β1 and collagen alpha1(I) mRNA (Canbay et al. 2003a, b).

Damaged hepatocytes also influence LSECs, which as an integral part of the hepatic reticulo-endothelial system have a role in HSC activation. LSECs are morphologically identified by their fenestrations, which are transcytoplasmic canals arranged in sieve plates. In healthy liver, this phenotype is maintained by hepatocytes and HSCs that release vascular endothelial growth factor (VEGF). Differentiated (i.e. fenestrated) LSECs prevent HSC activation and promote reversal of activated HSC to quiescence. However, upon liver injury, they lose this role. Preclinical studies have demonstrated that LSECs undergo defenestration as an early event that not only precedes liver fibrosis, but on its own may also promote it, proving that changes in LSEC differentiation might be an integral part in the development of fibrosis. In addition, during fibrogenesis, LSECs become highly pro-inflammatory and secrete an array of cytokines and chemokines (Connolly et al. 2010; DeLeve 2013; Ding et al. 2014; Xie et al. 2012, 2013).

## KE–KC activation and macrophage recruitment

KCs constitute 80–90 % of the tissue macrophages in the liver reticulo-endothelial system and account for approximately 15 % of the total liver cell population (Bouwens et al. 1986; Kolios et al. 2006). When activated, they are involved in pathogenesis of chemical-induced liver injury through the release of inflammatory mediators including cytokines, chemokines, lysosomal, and proteolytic enzymes and are a main source of TGF-β1 (Luckey and Petersen 2001; Winwood and Arthur 1993). In addition, latent TGF-β1 can be activated by KC-secreted matrix metalloproteinase 9 (MMP-9) (Friedman 2002; Kisseleva and Brenner 2008). Activated KCs also contribute to oxidative stress, which activates a variety of transcription factors, like nuclear factor κB (NF-κB) and peroxisome proliferator-activated receptor-gamma (PPAR-γ), leading to increased gene expression and production of growth factors, inflammatory cytokines, and chemokines. KCs express mitogens and chemoattractants for HSCs, such as TNF-α, interleukin-1 (IL-1), and monocyte chemoattractant protein-1 (MCP-1). They also induce the expression of PDGF receptors on HSCs, which enhances cell proliferation (Kolios et al. 2006). In addition to being pro-inflammatory molecules, expressed TNF-α, TNF-related apoptosis-inducing ligand (TRAIL), and Fas Ligand (FasL) are also capable of inducing death receptor-mediated apoptosis in hepatocytes (Kershenobich Stalnikowitz and Weissbrod 2003; Roberts et al. 2007). Under oxidative stress, macrophages are further activated leading to an enhanced inflammatory response that further activates KCs though cytokines [interferon gamma (IFNγ), granulocyte macrophage colony-stimulating factor (GM-CSF), TNF-α], bacterial lipopolysaccharides, extracellular matrix proteins, and other chemical mediators (Kershenobich Stalnikowitz and Weissbrod 2003).

Besides KCs, which are the resident hepatic macrophages, infiltrating bone marrow-derived macrophages, originating from circulating monocytes, are recruited to the injured liver via chemokine signals. KCs appear essential for sensing tissue injury and initiating inflammatory responses, while infiltrating Ly-6C+ monocyte-derived macrophages are more linked to chronic inflammation and fibrogenesis (Tacke and Zimmermann 2014). The relevance of KCs during chronic hepatic injury was demonstrated by blocking the infiltration of additional inflammatory monocytes via pharmacological inhibition of the chemokine CCL2 (Baeck et al. 2012). KC activation and macrophage recruitment are two separate events, both indispensable for fibrogenesis. Since they occur in parallel, they can be summarised as one KE (Pellicoro et al. 2014).

Probably there is a threshold for KC activation above which liver damage is induced. Pretreatment with gadolinium chloride, which inhibits KC function, resulted in reduced hepatocyte and LSEC injury, decreased number of macrophages in hepatic lesions and inhibited TGF-β1 mRNA expression in macrophages (Andres et al. 2003; Ide et al. 2005). Experimental inhibition of KC function or their depletion seemed to protect against chemical-induced liver injury, which supports the essentiality of this KE (Lotersztajn et al. 2005; Schumann et al. 2000).

### KER between KC activation and TGF-β1 expression

Following activation, KCs become the main source of TGF-β1, the most potent pro-fibrogenic cytokine, as well as of inflammatory mediators and ROS (Bataller and Brenner 2005; Brenner 2009; Guo and Friedman 2007; Kirkham 2007; Kolios et al. 2006; Lee and Friedman 2011). The experimental support for this KER came already in 1990 when it was demonstrated that KCs isolated from alcohol-induced fibrotic rat livers express and release TGF-β1, which was associated with KC-conditioned medium-induced stimulation of collagen formation by HSCs (Matsuoka and Tsukamoto 1990). Further confirmation came from observation that freshly isolated KCs have an increased mRNA expression of three acute phase cytokines (i.e. TNF-α, IL-6, and TGF-β) (Kamimura and Tsukamoto 1995) and that accumulated CD11b1 macrophages are critical for activating HSCs via expression of TGF-β1 (Chu et al. 2013).

## KE–TGF-β1 expression

TGF-β1 is a polypeptide member of the TGF-β superfamily of cytokines. TGF-β is synthezised as a non-active pro-form that forms a complex with both latency-associated protein (LAP) and latent TGF-β binding protein (LTBP) and undergoes proteolytic cleavage by the endopeptidase furin to generate the mature TGF-β dimer. Three TGF-β isoforms (i.e. β1, β2 and β3) have been identified, but only TGF-β1 was linked to liver fibrogenesis and is the most potent fibrogenic factor for HSCs (Gressner and Weiskirchen 2006). It plays a central role in fibrogenesis, mediating crosstalk between parenchymal, inflammatory, and collagen expressing cells. TGF-β1 is released by activated KCs, LSECs, and platelets. After activation HSCs themselves express TGF-β1. Hepatocytes do not produce TGF-β1, but are implicated in intracellular activation of latent TGF-β1. TGF-β1 can also induce its own mRNA expression to sustain high levels in local sites of liver injury (Kisseleva and Brenner 2008). TGF-β1 activates HSCs, stimulates extracellular matrix (ECM) synthesis, and suppresses its degradation. It stimulates collagen transcription in HSCs and the expression of connective tissue growth factor (CTGF), a pro-fibrogenic peptide that stimulates the synthesis of collagen type I and fibronectin; further it induces the expression of tissue inhibitor of metalloproteinases-1 (TIMP-1), an inhibitor of the collagen cleaving enzymes MMP-8 and MMP-13. TGF-β1 increases the α1(I) collagen mRNA half-life, mediated by increasing stability of α1(I) collagen mRNA through mitogen-activated protein kinases (MAPK) (Li et al. 2008). TGF-β1 further recruits inflammatory cells, portal fibroblasts, and circulating myofibroblasts to the injured liver and triggers the apoptosis of hepatocytes (Kershenobich Stalnikowitz and Weissbrod 2003; Leask and Abraham 2004; Parsons et al. 2007; Williams et al. 2000). TGF-β1 is an established mediator and regulator of epithelial–mesenchymal-transition (EMT—the process of transition of differentiated epithelial cells into mesenchymal cells), which further contributes to the production of ECM. It has been shown that TGF-β1 mediates EMT by inducing the transcription factor snail-1 and tyrosine phosphorylation of Smad2/3 with subsequent recruitment of Smad4 (Bataller and Brenner 2005; Brenner 2009; Gressner et al. 2002; Guo and Friedman 2007; Kaimori et al. 2007) (Fig. 3). Strategies aimed at disrupting TGF-β1 expression or signalling pathways are extensively being investigated since blocking this cytokine may not only inhibit ECM production, but also accelerate its degradation. Animal experiments using different strategies to block TGF-β1 have demonstrated significant anti-fibrotic effects. Inhibition of experimental fibrosis can be done by anti-TGF-β treatments with neutralising antibodies or soluble TbRs (TGF-β receptors) (Cheng et al. 2009; Liu et al. 2006; Lotersztajn et al. 2005; Qi et al. 1999; Tang et al. 2012).

### KER between TGF-β1 expression and HSC activation

TGF-β1 represents the most potent fibrogenic factor for HSCs, facilitating their transdifferentiation from quiescent vitamin A—storing cells to proliferative and contractile myofibroblasts. The effects of TGF-β1 are mediated by intracellular signalling via Smad proteins. Smads-2 and 3 are stimulatory, whereas Smad-7 acts inhibitory. Smad1/5/8, MAPK, and PI3 kinase drive further signalling pathways in different cell types for TGF-β1 effects (Bataller and Brenner 2005; Brenner 2009; Friedman 2002, 2008; Gressner et al. 2002; Kisseleva and Brenner 2007; Leask and Abraham 2004; Li et al. 2008; Liu et al. 2006; Parsons et al. 2007). Providing evidence for TGF-β1-induced HSC, activation in vitro is impeded by spontaneous activation of HSCs under these conditions. Furthermore, at the gene expression level, HSCs activated in in vitro conditions do not fully reproduce the changes observed in vivo (De Minicis et al. 2007). However, Czaja et al. (1989) did prove that treatment of cultured hepatic cells with TGF-β1 increased type I pro-collagen mRNA levels 13-fold due to posttranscriptional gene regulation and in Tan et al. (2013), it was shown that short TGF-β1 pulses can exert long-lasting effects on fibroblasts.

## KE–HSC activation

Multiple cells and cytokines play a role in the regulation of HSC activation, which consists of discrete responses—including proliferation, contractility, fibrogenesis, ECM degradation, chemotaxis, and retinoid loss (Friedman 2010). A two-phase process starts with the initiation phase, which is triggered by injured hepatocytes, ROS, and paracrine stimulation from neighbouring cell types (i.e. KCs, LSECs, and platelets), rendering HSCs sensitised to activation by up-regulating various receptors. The perpetuation phase refers to maintaining HSCs in an activated state, which is a dynamic process, including the secretion of autocrine and paracrine growth factors, such as TGF-β1, chemokines, and the up-regulation of collagen synthesis, mainly type I collagen. In response to growth factors, such as PDGF and VEGF, HSCs proliferate. Increased contractility leads to increased resistance to portal venous flow. Driven by chemoattractants, HSC accumulates in areas of injury. TGF-β1 synthesis promotes activation of neighbouring quiescent HSCs, whereas the release of hepatocyte growth factor (HGF) stimulates regeneration of adjacent hepatocytes. The release of chemoattractants like monocyte chemoattractant protein-1 (MCP-1) and colony-stimulating factors (CSFs) amplifies inflammation. Activated HSCs (i.e. myofibroblasts) are the primary collagen producing cells, the key cellular mediators of fibrosis, and a nexus for converging inflammatory pathways leading to fibrosis (Bataller and Brenner 2005; Friedman 2002, 2004; Kisseleva and Brenner 2007; Lee and Friedman 2011; Li et al. 2008) (Fig. 3). Experimental inhibition of HSC activation prevents fibrosis (Nakamura et al. 2014; Son et al. 2009), which led to the development of anti-fibrotic therapeutic strategies that include inhibition of HSC proliferation or stimulation of HSC apoptosis (Anan et al. 2006; Li et al. 2008; Lotersztajn et al. 2005).

### KER between HSC activation and collagen accumulation/changes in ECM composition

Up-regulation of collagen, mainly type I, synthesis following HSC activation is among the most striking molecular responses of HSCs to injury and is mediated by both transcriptional and posttranscriptional mechanisms. The half-life of collagen α1(I) mRNA increases 20-fold in activated HSCs compared to quiescent HSCs (Li et al. 2008). Together with decreased matrix degradation (expression of degrading MMPs is down-regulated while their inhibitors TIMPs are up-regulated), ECM composition changes and further stimulates HSC activation and production of TGF-β1. Also, increased mechanical stiffness of the ECM activates HSCs through integrin signalling (Benyon and Arthur 2001; Lee and Friedman 2011; Milani et al. 1994). Monocytes and macrophages are involved in inflammatory actions by producing large amounts of nitric oxide (NO) and inflammatory cytokines, such as TNF-α, which have a direct stimulatory effect on HSC collagen synthesis. Chronic inflammation, hypoxia, and oxidative stress reactivate EMT developmental programmes that converge in the activation of NF-κB (Kershenobich Stalnikowitz and Weissbrod 2003; Lopez-Novoa and Nieto 2009; Thompson et al. 2011). Since it is difficult to stimulate sufficient collagen production and its subsequent incorporation into a pericellular matrix in vitro, analytical methods were developed to measure pro-collagen secreted into culture medium or α-SMA expression, a marker of fibroblast activation. In primary culture, HSCs from normal liver begin to express α-SMA coincident with culture-induced activation (Brenner 2009; Chen and Raghunath 2009; Rockey et al. 1992; Yin et al. 2013).

## KE: collagen accumulation and changes in ECM composition

Irrespective of upstream events that trigger and maintain fibrosis, the final product of myofibroblast cellular activity is the massive deposition of collagen, which results in fibrosis (Friedman 2003). The overall amount of collagen deposited by fibroblasts is a result of regulated balance between collagen synthesis and collagen catabolism. HSCs generate fibrosis not only by increasing cell number, but also by increasing ECM production per cell. The basement membrane-like ECM normally consists of collagens IV and VI, which is progressively replaced by collagens I and III as well as cellular fibronectin during fibrogenesis (Gressner and Weiskirchen 2006; Kisseleva and Brenner 2008; Lotersztajn et al. 2005). Although multiple ECM components are tremendously up-regulated in hepatic fibrosis, type I collagen is the most abundant one. These changes in ECM composition initiate several positive feedback pathways that further amplify fibrosis. Increasing ECM stiffness is a stimulus for HSC activation (Lee and Friedman 2011). ECM-provoked signals link with other growth factor receptors through integrin-linked kinase and transduce signals to the actin cytoskeleton that promote migration and contraction (via membrane-bound guanosine triphosphate binding proteins, in particular Rho67 and Rac). Activation of cellular matrix MMPs leads to a release of growth factors from ECM-bound reservoirs in the extracellular space, which further stimulates cellular growth and fibrogenesis (Milani et al. 1994).

In addition to a transition of quiescent HSCs into activated HSCs and then further into contractile myofibroblasts, other cells may transdifferentiate into fibrogenic myofibroblasts in liver injury. Additional sources of ECM include bone marrow, portal fibroblasts, EMT from hepatocytes, and cholangiocytes (Henderson and Iredale 2007). Therefore, continuing imbalance between the deposition and degradation of the extracellular matrix is a prerequisite of liver fibrosis and therefore essential for the AO (Bataller and Brenner 2005).

### KER between collagen accumulation/changes in ECM composition and liver fibrosis

There is a smooth transition without a definite threshold from ECM accumulation to liver fibrosis, which is characterised by distortion of the normal hepatic architecture through formation of fibrous scars. There is plenty of in vivo evidence that ECM accumulation is a prestage of liver fibrosis (Bataller and Brenner 2005; Brancatelli et al. 2009; Lee and Friedman 2011; Pellicoro et al. 2014; Poynard et al. 1997; Rockey and Friedman 2006).

## Additional pro-fibrogenic actors

Couple of other actors could play an important role in driving fibrogenesis without being labelled KEs. Chronic inflammation and oxidative stress are ongoing processes throughout the pathway and mutually interconnected with most of the KEs, being contributors to, as well as, consequences of the on-going fibrogenic process (Kershenobich Stalnikowitz and Weissbrod 2003; Parola and Robino 2001; Sanchez-Valle et al. 2012; Sivakumar and Das 2008). In addition, there are some important fibrogenic signalling pathways that influence HSC activation and fibrogenesis, such as those belonging to adipokine–leptin system, neuroendocrine pathways, and renin–angiotensin system.

### Oxidative stress

Oxidative stress is reflected in an imbalance between the rate of oxidant production and its degradation. It plays a crucial role in liver fibrogenesis by inducing hepatocyte injury and death, by activating KCs and HSCs and by modulating both the expression and the activity of pro-fibrogenic cytokines (Kirkham 2007; Poli 2000; Singh and Czaja 2007) (Fig. 3). Hence, ROS likely contributes to both onset and progression of fibrosis, being simultaneously a contributor to and a consequence of the observed condition (El Rigal et al. 2013; Parola and Robino 2001). Oxidative stress-related molecules, including superoxide, hydrogen peroxide, hydroxyl radicals, and aldehydic end products, may be derived from hepatocytes, as well as from activated KCs, other inflammatory cells and HSCs (Kisseleva and Brenner 2007; Lee and Friedman 2011; Natarajan et al. 2006). Oxidative stress can activate a variety of transcription factors such as NF-κB and PPAR-γ, which may further lead to increased gene expression and production of growth factors, inflammatory cytokines, and chemokines, thus further fuelling inflammation (Parsons et al. 2007; Reuter et al. 2010). Antioxidants display anti-fibrogenic properties in cell cultures and in experimental animal models (Lotersztajn et al. 2005). Specifically, PDGF-induced increases in collagen deposition and liver fibrosis is markedly reduced by treatment with the anti-oxidant MnTBAP chloride, a cell permeable superoxide dismutase (SOD) mimetic and peroxynitrite scavenger (El Rigal et al. 2013).

### Chronic inflammation

Development of liver fibrosis is also driven by chronic inflammation in response to injury affecting all cell types involved in the pathogenesis. The fibrogenic cascade is maintained by mediators secreted by inflammatory and pro-fibrogenic cells that stimulate each other in amplifying the pathogenic process (Fig. 3). Damaged hepatocytes release inflammatory cytokines that activate macrophages (Kupffer cells) and stimulate further recruitment of inflammatory cells, which produce pro-fibrotic cytokines and chemokines that in turn activate fibroblasts (Fujiwara and Kobayashi 2005). Dead or apoptotic hepatocytes are phagocytosed by leukocytes resulting in release of pro-inflammatory cytokines (TNF, IL-6 and IL-1β) and recruitment of T cells (Bataller and Brenner 2005). Similarly, resident Kupffer cells secrete various cytokines and chemokines upon their activation (TGFβ, CCL2 and CCL5; Tacke and Zimmermann 2014). However, the central role belongs to activated HSCs, of which various functions include direct interaction with different immune cell populations, secretion of pro-inflammatory cytokines and chemokines (such as CCL2-5, CCR5, CCR7), production of NOX enzymes and ROS, as well as presenting antigens in the injured liver. All these properties allow us to consider stellate cells, central to the fibrogenic process, as innate immune cells (Pellicoro et al. 2014).

Recently, it became evident that also adaptive immune cells are involved in fibrogenesis (Xu et al. 2012), and moreover, it was suggested that the ratio of anti-fibrotic (T_H_1) and pro-fibrotic (T_H_2) T helper cells could influence the outcome of the fibrotic response, as observed in mouse models for fibrosis (Shi et al. 1997). T_H_2 cells secrete IL-13, a pro-fibrogenic mediator, which can promote fibrosis either by stimulating TGF-β1 synthesis and activation, or independently of TGF-β1, by controlling the relative expression of IL-13 receptors on myofibroblasts (Chiaramonte et al. 1999; Wynn 2004)). On the other hand, T_H_1 cells stimulate the production of anti-fibrotic mediators IFNγ and IL-12 (Muhanna et al. 2008). Additional T cell inflammatory mediators, whose roles in fibrogenesis still need to be confirmed, could be T_H_17 cells that were associated with induction of secretion of pro-inflammatory cytokines IL-1β, IL-6, TNF, and TGF-β by various cell types resident in liver (Korn et al. 2009); regulatory T cells (T_Reg_) that have exhibited both anti-fibrogenic (Katz et al. 2011) and pro-fibrogenic (Langhans et al. 2013) behaviour; and cytotoxic T cells (CD8^+^ T cells) that could serve a pro-fibrogenic role (Safadi et al. 2004), even though mice deficient in these cells showed no difference in the development of liver fibrosis upon CCl_4_ exposure (Novobrantseva et al. 2005). Interestingly, anti-fibrotic activity was also attributed to natural killer (NK) cells and γδ T cells that induce apoptosis of stellate cells (Hammerich et al. 2014; Taimr et al. 2003); whereas the natural killer T cells (NKT) were shown to promote fibrosis by secreting pro-fibrotic cytokines IL-4 and IL-13 (Bonecchi et al. 2000). In a mouse model of fibrosis, population of dendritic cells (DCs) increased significantly stimulating the activity of NK cells, T cells, and HSCs (Connolly et al. 2009). The complement system, as an innate immune mechanism of host defence, plays a role in fibrogenesis via the complement factor 5 (C5) (Hillebrandt et al. 2005), whose depletion in mice leads to impaired liver regeneration (Mastellos et al. 2001). Innate lymphoid cells (nuocytes), neutrophils, and mast cells are additional cell types that might play a role in fibrogenesis (Franceschini et al. 2007; Harty et al. 2010; Liang et al. 2013), even though the results are not always conclusive (Saito et al. 2003; Sugihara et al. 1999; Xu et al. 2004). Importantly, since the majority of findings discussed here comes from rodent models, additional research is needed to attribute these functions to human counterparts.

Chronic inflammatory response often goes hand in hand with tissue destruction and repair. Tissue damage is enhanced by activated inflammatory cells, which also represent a major source of oxidative stress-related molecules (Bataller and Brenner 2005; Henderson and Iredale 2007; Marra 2002; Parola and Robino 2001). Interestingly, suppression of inflammatory activity by eliminating the aetiological agent, such as a virus, or dampening the immune response can halt or even reverse the fibrotic process (Czaja 2014). Studies examining the role of individual inflammatory cell populations in experimental models provide evidence that the immune system can regulate the progression as well as the homeostasis or even regression of liver fibrosis (Pellicoro et al. 2014). Most probably, the outcome will depend on the aetiology that drives the fibrotic process as well as the balance between pro-fibrotic and anti-fibrotic cell elements, including populations of pro-inflammatory and pro-resolution macrophages, T helper cells, and non-conventional T cell subsets.

### Adipokine–leptin system

Adipokines are secreted mainly by adipose tissue, but also by resident and infiltrating macrophages and are increasingly recognised as mediators of fibrogenesis (Friedman 2010). Leptin promotes HSC fibrogenesis, enhances TIMP-1 expression and acts as a pro-fibrotic agent through suppression of PPARγ, an anti-fibrogenic nuclear receptor that can reverse HSC activation. The expression of leptin receptor is up-regulated during HSC activation; therefore, leptin activity is increased through enhanced signalling. Downstream effects include increased release of TGF-β1 from KCs. The counter-regulatory hormone adiponectin is reduced in hepatic fibrosis (Lee and Friedman 2011; Lotersztajn et al. 2005).

### Neuroendocrine pathways

Upon chronic liver injury, the local neuroendocrine system is triggered and activated HSCs express specific receptors, most prominently those regulating cannabinoid signalling. Activated HSCs are additionally a key source of the endogenous cannabinoid, 2-arachidonylglycerol (2-AG), which drives increased (cannabinoid-receptor) CB1 signalling. Stimulation of the CB1 receptor is pro-fibrogenic, whereas the CB2 receptor is anti-fibrotic and hepatoprotective. Opioid signalling increases proliferation and collagen production in HSCs. Serotonin has a pro-fibrotic effect that synergises with PDGF signalling. Also thyroid hormones enhance activation of HSC (through increased p75 neurotrophin receptor (p75NTR) and activation of Rho), thereby accelerating the development of liver fibrosis (Friedman 2010; Lee and Friedman 2011; Lotersztajn et al. 2005).

### Renin–angiotensin system

Angiotensin II (Ang II) is a pro-oxidant and fibrogenic cytokine that stimulates DNA synthesis, cell migration, procollagen α1(I) mRNA expression, and secretion of TGF-β1 and inflammatory cytokines. These fibrogenic actions are mediated by NOX (Kisseleva and Brenner 2007; Lee and Friedman 2011; Lotersztajn et al. 2005).

## Overall assessment of the AOP for liver fibrosis

Essentiality, biological plausibility and empirical evidence are explained in detail within respective KE and KER chapters and summarised in Table 1. We rated essentiality as “high” for all KEs due to the existence of experimental evidence that blocking them would prevent or attenuate, where complete blocking is not possible, the next downstream KE and therefore the whole AOP. Some evidence arises from preclinical research for anti-fibrotic agents, which is mainly based on the interference with a KE. In summary, pharmacological inhibition of liver cell apoptosis attenuates liver injury and fibrosis; experimental inhibition of KC function protects against liver injury and inhibits TGF-β1 mRNA expression in macrophages; animal experiments using different strategies to block TGF-β1 have demonstrated significant anti-fibrotic effects; and experimental inhibition of HSC activation prevents fibrosis.

Biological plausibility for the first KER (protein alkylation—cell injury) was rated as “moderate”, whereas all others were rated as “high”, because there is good scientific understanding of these relationships. On the other hand, empirical support for KERs was rated as “moderate”, because there is only limited empirical evidence that a change in the upstream KE leads to an appropriate change in the respective downstream KE, especially in respect to dose–response (KE_up_ occurring at lower dose than KE_down_), temporality (KE_up_ occurring at earlier time point than KE_down_), and incidence (KE_up_ with higher incidence than KE_down_). Due to the limited availability of adequate cell models, dose–response data on KERs are not available. Only in the case of the last KER (collagen accumulation-liver fibrosis), empirical support was considered as “high” due to sufficient amount of empirical and clinical evidence.

### Dealing with uncertainties and knowledge gaps

Covalent protein alkylation is a broad, non-specific MIE and a feature of many hepatotoxic drugs. However, the overall extent of protein binding does not adequately distinguish toxic from non-toxic effects (Bauman et al. 2009). Some chemicals significantly alkylate proteins without causing toxicity, which suggests that only alkylation of specific protein subsets contributes to injury. Indeed, Codreanu et al. (2014) presented an inventory of proteins affected by electrophile-mediated alkylation in intact cells and suggested that non-toxic covalent binding largely affects cytoskeletal protein components, whereas toxic covalent binding induces lethal injury by targeting factors involved in protein synthesis and catabolism and possibly mitochondrial electron transport. Future studies including toxic and non-toxic drugs could test these hypotheses and provide a better mechanistic basis for interpreting protein alkylation in drug safety evaluation. The identification and specification of the targeted biomolecules would allow the structural definition of chemical initiators and consecutively the profiling and categorising of chemicals related to the initiation of this AOP. Furthermore, it is unknown whether there is a threshold for initiating this pathway and whether this threshold would relate to the extent of alkylation of a single or numerous proteins. DNA alkylation (methylation) could play a role as well, but for the time being there is no data to substantiate this claim.

By definition, an AOP has only one MIE and one final AO, which constitute the two anchor points. Any other MIE that leads to cell injury and further to liver fibrosis via the same downstream KEs would constitutes another AOP (OECD 2013). However, different agents that cause hepatocyte injury by various MIEs would finally lead to fibrosis via the same described downstream KEs. E.g. the fibrogenic drug methotrexate binds to the enzyme dihydrofolate reductase as first molecular interaction (Jennings et al. 2014). Hepatocyte injury, therefore, is an early convergent KE for several AOPs, proving its essentiality for fibrogenesis. Still, hepatocyte injury does not inevitably lead to fibrosis; there are hepatotoxic chemicals, like acetaminophen (http://livertox.nih.gov/Acetaminophen.htm) for which liver fibrosis has not been observed. The difference in progression to liver fibrosis might lie in various cellular responses such as apoptosis, necrosis, transdifferentiation, or repair and regeneration. There is increasing evidence for apoptosis being a main fibrogenic trigger (Canbay et al. 2004a, b; Wang et al. 2013). Hepatocyte insult/injury without cell death might already be sufficient to trigger fibrosis. Potentially, fibrosis-specific features of cell injury could be the amount (quantitative difference) rather than the kind (qualitative difference) of cell injury. The rate of cell injury/death, i.e. the amount of injury within a certain time frame could be another plausible initiating parameter, as fibrosis is resulting from chronic injury. Assuming hepatocyte injury represents a crucial KE, without which fibrosis could not occur via this AOP, a simple investigation of in vitro hepatotoxicity could provide relevant information for potential fibrosis prediction without the need of a highly elaborate cell models. EMT, the process of transition of differentiated epithelial cells into mesenchymal cells, is an additional contribution to fibrogenesis and could be a potential fibrosis-specific marker for hepatocytes experiencing injury. This concept is still controversial and discussions are on-going (Blachier et al. 2013; Iwaisako et al. 2012; Kisseleva and Brenner 2011; Liedtke et al. 2013; Scholten and Weiskirchen 2011; Wells 2010; Zeisberg et al. 2007).

## Discussion

Liver fibrosis is an important health issue with clear regulatory relevance. Progressive hepatic fibrosis, ultimately leading to cirrhosis, is a significant contributor to global health burden (Lim and Kim 2008). In the European Union, 0.1 % of the population is affected by cirrhosis, the most advanced stage of liver fibrosis with full architectural disturbances (Van Agthoven et al. 2001). Besides the epidemiological relevance, liver fibrosis also imposes a considerable economic burden on society. Indeed, the only curative therapy for chronic liver failure is liver transplantation. More than 5.500 orthotopic liver transplantations are currently performed in Europe on a yearly basis, costing up to €100.000 the first year and €10.000 yearly thereafter (Safadi and Friedman 2002). Therefore, much effort is put in research to find therapeutic strategies. Several targets for anti-fibrotic agents have been identified—first and foremost myofibroblasts, but also fibrogenic cytokines, their receptors and signalling pathways (Gressner et al. 2009; Gressner and Weiskirchen 2006; Li et al. 2008; Lotersztajn et al. 2005; Schuppan and Kim 2013). Even the inhibition of hepatocyte apoptosis has been considered. These approaches are also helpful for the identification of KEs and their essentiality for the AO. Unfortunately, none of these strategies has proved applicable for clinical interventions because of their severe adverse effects. Research is further complicated by the lack of sensitive and specific clinical biomarkers to measure fibrosis progression or reversal (Friedman 2010).

An alternative approach, which deals with prevention rather than with consequences of a diseased state, emerges from an AOP concept. It aims at using mechanistic toxicological information in order to devise novel strategies for prediction of chemical toxicity. This AOP description is among the first AOPs that have been developed according to the OECD guidelines; it has been repeatedly revised along with the evolving practice and guidance in AOP development based on increasing experience. It is a plausible qualitative description of the association between AO and MIE across different levels of biological organisation, based on reviews and published data from in vivo, in vitro data, also including available data from clinical observations. The collection of mechanistic data turned out to be rather challenging. Animal studies are mainly focused on the AO (apical endpoint) and rarely describe mechanistic sequences in detail. Single cell cultures of various liver cell types allow studying the individual cell responses to injury and provide the opportunity to understand the roles that different liver cell types play in fibrogenic processes. Several co-culture models allow the investigation of interactions between some individual actors in vitro. But in general, these studies were not designed to investigate the linkages between various actors and on the whole it is rather difficult to find sound empirical evidence to support KERs. Due to the pathogenic complexity of liver fibrosis with the involvement of many different cell types, there is currently no suitable cell model available to challenge the reliability and robustness of the AOP and to mimic and further explore the sequence of events, especially in quantitative terms. The investigation of quantitative aspects regarding how much change for how long in an upstream KE is needed to cause a corresponding change in the next downstream KE remains an ambitious goal.

**Fig. 4:**
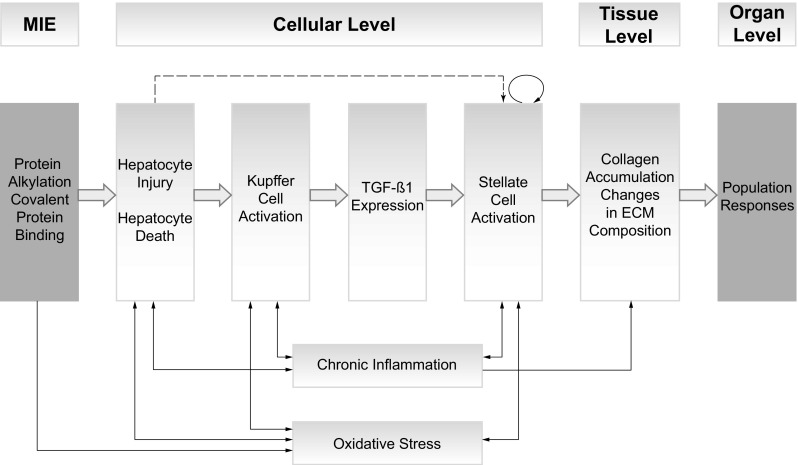
Graphic representation of the adverse outcome pathway describing the linkage between hepatic injury caused by protein alkylation and the formation of liver fibrosis, including the display of chronic inflammation and oxidative stress, thus illustrating their interrelationships with the various KEs

Two actors have been highlighted as essential contributors to fibrogenesis without being labelled KEs, namely chronic inflammation and oxidative stress, which are ongoing processes throughout the pathway and mutually interconnected with most of the KEs (Kershenobich Stalnikowitz and Weissbrod 2003; Parola and Robino 2001; Sanchez-Valle et al. 2012; Sivakumar and Das 2008). It has been extensively and repeatedly discussed with the AOP developer community how to best insert these events into the AOP. As they are interrelated with all the other KEs, they cannot be put at a certain position inside the pathway (which, by definition, does not allow any branches or bypasses). On the other hand, the removal from the overview with their only mention in the individual KEs and KERs descriptions does not adequately reflect their important role in fibrogenesis. A valid solution has still to be found and would also benefit other AOPs, which face similar problems. For illustrative reasons, Fig. 4 shows these interrelationships.

The complex mechanism of fibrogenesis does not only affect a single organ, but causes a systemic response which equally damages other organs and tissues. The described findings in liver fibrosis parallel those in studies of fibrogenesis in other organs. The same kind of cells and soluble factors is involved in all organs (Friedman 2010), e.g. the reference compound CCl_4_ equally affects lymphoid organs, lungs, and kidneys (Kisseleva and Brenner 2008). Fibrosis may affect lung, kidney, heart and blood vessels, eye, skin, pancreas, intestine, brain, and bone marrow. Furthermore, multi-organ fibrosis could occur due to mechanical injury or could be drug- or radiation-induced (Liu 2011; Wynn 2007, 2008). Since many fibrogenic pathways are conserved across tissues, recent findings in liver might be extended to studies of fibrosis in lungs, kidneys, heart, and other organs. Identified targets for anti-fibrotic therapeutic agents could therefore be valid for all organs that are susceptible to fibrosis (Sivakumar and Das 2008). Importantly, this AOP is not restrictive regarding sex and life-stage.

In addition, findings suggest common conserved pathways across different species which initiate and promote liver fibrosis. Animal models are used to study fibrogenesis and CCl_4_ intoxication in rats, and mice is probably the most widely studied and therefore best characterised model with respect to histological, biochemical, cellular, and molecular changes associated with the development of fibrosis (Constandinou et al. 2005; Iredale 2007).

## Conclusion

AOP methodology provides a framework for collecting, organising, and evaluating relevant mechanistic information on the toxic effects of chemicals, and it is still evolving with increasing experience from the growing community of AOP developers. AOP development is an iterative and dynamic process with continuous expansion in accordance with increasing scientific knowledge. The described available mechanistic toxicological information serves as a knowledge-based repository for the identification of KEs along the pathway and might be helpful for the identification of novel biomarkers. In vitro methods for measuring these KEs, KERs, and biomarkers can be selected or developed to ultimately support the prediction of chemical toxicity. Early (upstream) markers for downstream events can be identified to facilitate a testing strategy for chemical risk assessment. In addition, the KEs can be used for hazard identification and read-across to assess the toxic potential of an untested substance. Therefore, AOPs have the potential to become a powerful tool to support alternative methods for chemical risk assessment which may be predictive of the AO in vivo without the need to actually demonstrate the AO. Further AOP development will be facilitated by the AOP Wiki, which provides a platform for interdisciplinary collaboration between the scientists and the regulators. It is essential that many researchers from various disciplines like toxicology, biology, chemistry, clinical medicine, and computer modelling engage in this activity and contribute to AOP development. AOP developers need to share and connect their AOPs through common KEs to build AOP networks that eventually will better represent the complex biological processes and interactions in response to various chemical exposures.

This AOP description demonstrates how detailed and distributed mechanistic information from the literature can be assembled and coherently displayed to support the use of alternative data. Together with other AOP descriptions leading to chronic liver injury, it provided the basis for the design of feasibility studies for predicting selected types of liver toxicity within the SEURAT-1 research project. The addition of quantitative data on dose–response relationships, threshold values, and temporal sequences, the acquisition of which is dependent on the availability of suitable cell models, would substantially improve the applicability of this AOP.
